# Global Climate and Human Health Effects of the Gasoline and Diesel Vehicle Fleets

**DOI:** 10.1029/2019GH000240

**Published:** 2020-03-11

**Authors:** Yaoxian Huang, Nadine Unger, Kandice Harper, Chris Heyes

**Affiliations:** ^1^ Department of Civil and Environmental Engineering Wayne State University Detroit MI USA; ^2^ College of Engineering, Mathematics, and Physical Sciences University of Exeter Exeter UK; ^3^ School of Forestry and Environmental Studies Yale University New Haven CT USA; ^4^ International Institute for Applied Systems Analysis Laxenburg Austria

**Keywords:** gasoline and diesel, climate change, premature deaths

## Abstract

The global gasoline and diesel fuel vehicle fleets impose substantial impacts on air quality, human health, and climate change. Here we quantify the global radiative forcing and human health impacts of the global gasoline and diesel sectors using the NCAR CESM global chemistry‐climate model for year 2015 emissions from the IIASA GAINS inventory. Net global radiative effects of short‐lived climate forcers (including aerosols, ozone, and methane) from the gasoline and diesel sectors are +13.6 and +9.4 mW m^−2^, respectively. The annual mean net aerosol contributions to the net radiative effects of gasoline and diesel are −9.6 ± 2.0 and +8.8 ± 5.8 mW m^−2^. Aerosol indirect effects for the gasoline and diesel road vehicle sectors are −16.6 ± 2.1 and −40.6 ± 4.0 mW m^−2^. The fractional contributions of short‐lived climate forcers to the total global climate impact including carbon dioxide on the 20‐year time scale are similar, 14.9% and 14.4% for gasoline and diesel, respectively. Global annual total PM_2.5_‐ and ozone‐induced premature deaths for gasoline and diesel sectors approach 115,000 (95% CI: 69,000–153,600) and 122,100 (95% CI: 78,500–157,500), with corresponding years of life lost of 2.10 (95% CI: 1.23–2.66) and 2.21 (95% CI: 1.47–2.85) million years. Substantial regional variability of premature death rates is found for the diesel sector when the regional health effects are normalized by the annual total regional vehicle distance traveled. Regional premature death rates for the gasoline and diesel sectors, respectively, vary by a factor of eight and two orders of magnitude, with India showing the highest for both gasoline and diesel sectors.

## Key Points

- The net radiative effects for the gasoline and diesel sectors over a 20‐year time scale are +91.4 and +65.7 mW m^−2^, respectively
- Global annual total premature deaths of 115,000 and 122,100 are attributable to the gasoline and diesel sectors
- There exists substantial regional variability of premature death rates for the diesel sector

## 1 Introduction

The global road transportation sector is a major contributor to emissions of both long‐lived greenhouse gases (e.g., carbon dioxide, CO_2_) and short‐lived climate forcers (SLCFs), including aerosols (Rönkkö et al., 2017) and precursors of ozone (O_3_). SLCFs affect air quality (Matthes et al., 2007; Niemeier et al., 2006), human health (Anenberg et al., 2017, 2019; Barrett et al., 2015; Hill et al., 2009; Jacobson, 2007; Shindell et al., 2011), the atmospheric oxidation capacity (Ban‐Weiss et al., 2008; Hoor et al., 2009), and climate change (Balkanski et al., 2010; Fuglestvedt et al., 2008, 2010; Unger et al., 2009). Reduction of transportation emissions plays a critical role in achieving the 1.5 °C goal of the Paris Agreement (Masson‐Delmotte et al., 2018). Therefore, the potential scope of mitigation in this sector needs to be assessed.

Emitted SLCFs from gasoline vehicles include nonmethane volatile organic compounds (NMVOCs) and carbon monoxide (CO), with important implications for surface O_3_ formation (Granier & Brasseur, 2003; Huang et al., 2017) and secondary organic aerosol production (Kanakidou et al., 2005). In contrast, a large amount of black carbon (BC) and nitrogen oxides (NO_x_) are emitted from diesel vehicles (Ban‐Weiss et al., 2008; Lund et al., 2014). In recent years, it has emerged that diesel vehicles emit 4–7 times more NO_x_ in real‐world driving conditions compared to laboratory approval tests, the so‐called “excess NO_x_” (Anenberg et al., 2017), leading to concerns for air quality and adverse human health impacts (Anenberg et al., 2019; Holland et al., 2016; Hou et al., 2016). Different regions have diverse penetrations of gasoline and diesel fuels in their on‐road vehicle fleets. For instance, most of the light‐duty motor vehicles in the United States are fueled by gasoline (Ban‐Weiss et al., 2008), while about 40% of the passenger vehicles and most heavy duty trucks and buses in Europe are powered by diesel fuels (Anenberg et al., 2019). The different fuel‐type composition of vehicle fleets causes distinctive spatial variability in SLCF emissions, potentially leading to different impacts on surface air quality associated with surface PM_2.5_ (particulate matter with aerodynamic diameter equal to or less than 2.5 micrometers) and O_3_ pollution levels.

From a climate impact perspective, the on‐road transportation sector has been estimated to have contributed a historical net radiative forcing of +178 mW m^−2^ between preindustrial and present day (Fuglestvedt et al., 2008), and has been ranked the most warming economic sector on short time scales (20–30 years) (Unger et al., 2010). For year 2000 emissions, the net radiative forcing of on‐road transportation emissions (including CO_2_ and SLCFs) has been estimated to be +199 and +477 mW m^−2^ for the 20‐ and 100‐year time horizons, respectively (Unger et al., 2010). A previous assessment suggests a net SLCF radiative forcing for the global diesel vehicle fleet of +28 mW m^−2^ for year 2010 emissions (Lund et al., 2014). From an air quality perspective, surface PM_2.5_ and O_3_ pollution is associated with cardiovascular disease and lung cancer, leading to premature death (Stanaway et al., 2018). Previous studies indicate that on‐road transportation sector emissions are a major contributor to elevated surface PM_2.5_ and O_3_ concentrations (Granier & Brasseur, 2003; Yan et al., 2011), which are associated with approximately 165,000–385,000 human premature deaths per year (Anenberg et al., 2019; Chambliss et al., 2014; Lelieveld et al., 2015; Silva et al., 2016). Because of the warming BC emissions and excess NO_x_, diesel has typically received more attention than gasoline. A comparative assessment of the climate and health impacts of both fuel types is needed.

In this study, we employ a global chemistry‐climate model, the NCAR Community Earth System Model (CESM) CAM5‐Chem (Community Atmosphere Model version 5.5 coupled with chemistry), to quantify the impacts of the global gasoline and diesel vehicle fleet emissions on air quality, climate, and public health. Section 2 describes the methodology including the calculations of the radiative effects from SLCFs and the premature deaths associated with PM_2.5_ and O_3_ from gasoline and diesel emissions. Results and discussion are presented in section 3. We present conclusions in section 4.

## 2 Methods

### 2.1 CAM5‐Chem Model Simulations

We employ the CAM5‐Chem model in CESM version 1.2.2 to investigate the impacts of global gasoline and diesel emissions on air quality, climate, and public health (Emmons et al., 2010; Huang et al., 2018; Lamarque et al., 2012; Tilmes et al., 2015). CAM5‐Chem contains a coupled NO_x_‐VOC‐Ozone‐Aerosol chemistry scheme, with horizontal resolution of 0.9° latitude by 1.25° longitude and 56 vertical levels from the surface up to about 40 km. Sea surface temperature and sea ice in the model are prescribed, which are from the Climatological/Slab‐Ocean Data Model (DOCN) and Climatological Ice Model (DICE), respectively. CAM5‐Chem is driven by offline GEOS‐5 (Goddard Earth Observing System model version 5) meteorological fields.

Global spatially gridded anthropogenic emissions are from the International Institute for Applied Systems Analysis (IIASA) Greenhouse Gas‐Air Pollution Interactions and Synergies (GAINS) ECLIPSE V5a (Evaluating the Climate and Air Quality Impacts of Short‐lived Pollutants version 5a) inventory for the year 2015 (Amann et al., 2011, 2013; Klimont et al., 2017; Stohl et al., 2015). The global and regional annual anthropogenic emission budgets for the year 2015 IIASA GAINS ECLIPSE V5a inventory and the contributions from the on‐road gasoline and diesel sectors are shown in Table 1. For each fuel type, it includes emissions from all light‐ and heavy‐duty vehicle types, such as cars, vans, trucks, and buses. The model configuration of this study is identical to Huang et al. (2018) that provided an evaluation of model performance for BC, organic aerosols, and aerosol optical depth against multiple observational data sets. This study applies the 3‐mode modal aerosol module to represent microphysical process of aerosols (Liu et al., 2012).

**Table 1:** Annual Global and Regional Anthropogenic Emissions of Species From ECLIPSE V5a Inventory and Separately From Gasoline and Diesel Sectors in ECLIPSE V5a for the Year 2015 (Units: kt species year^−1^)

Specie	ECLIPSE V5a					Gasoline					Diesel				
	Global	USA	Europe	China	India	Global	USA	Europe	China	India	Global	USA	Europe	China	India
BC	6,757	184.6	286.7	1,662	1,514	148.5	19.9	1.26	41.3	6.99	952.7	37.7	65.5	148.5	114.8
POM	12,378	307.4	414.6	2,867	2,795	316.3	25.2	4	58	20.8	467.8	24	79	124	79
SO_2_	90,795	6,283	3,650	23,602	12,433	238.4	8.29	1.73	14.1	25.3	372.3	2.18	4.37	27.2	84
NO_x_	114,113	11,489	7,543	22,792	8,600	8,780	1,657	214	1,148	247	22,772	2,041	3,088	4,269	2,354
NMVOCs	108,840	8,627	6,648	24,367	13,039	22,054	3,440	695	3,104	1,150	1,690	180	126	289	175
CO	524,738	34,071	20,062	166,291	70,545	115,955	17,073	3,641	16,693	5,528	16,435	1,965	736.6	3,659	1,814
NH_3_	57,902	3,796	3,920	15,253	10,978	475.2	126.2	56	70.8	13	21.6	1.81	13.1	1.02	0.59
CH_4_	330,991	23,554	17,845	55,909	41,168	1,184	266	33.1	145.6	55.4	333.5	72.6	10.6	49	24.5

A control simulation is performed with the anthropogenic emission inventory from ECLIPSE V5a along with two sensitivity simulations in which the gasoline and diesel road vehicle emissions are removed, respectively. All simulations are run for 6 years with the first year discarded as spin up and the last five years of model output data averaged for analysis. The contributions of the gasoline and diesel sectors to surface air quality and radiative forcing are then determined by taking the difference between the control and relevant sensitivity simulation.

### 2.2 SLCF Radiative Forcing Calculations

The aerosol radiative effects are calculated online in CAM5‐Chem using the Rapid Radiative Transfer Model (Iacono et al., 2008). Aerosol radiative effects include the direct and indirect radiative effects as well as the surface albedo effect (Ghan, 2013; Ghan et al., 2012). Indirect radiative effect comprises of the first (albedo), second (lifetime), and the semidirect effects. The net impacts of the short‐lived emission precursors (CO, NMVOCs, and NO_x_) on O_3_ and CH_4_ radiative forcing, including direct emissions, indirect effects on chemical production and lifetime, and stratospheric water vapor, are assessed using the 20‐year time horizon global warming potential climate policy metrics from the Fifth Assessment Report of the Intergovernmental Panel on Climate Change (IPCC; Myhre et al., 2013) and Collins et al. (2013).

### 2.3 PM_2.5_‐ and O_3_‐Induced Premature Deaths Calculation

PM_2.5_‐induced premature deaths are estimated following previous studies (Anenberg et al., 2017; Apte et al., 2015), with integrated exposure responses data set using the Global Burden of Disease 2017 study (Stanaway et al., 2018, hereafter referred to as GBD2017). We calculate health endpoints for diseases including children's (<5 years) acute lower respiratory infection (ALRI); adult (>25 years) chronic obstructive pulmonary disease (COPD), lung cancer (LC), ischemic heart disease (IHD), and stroke. The premature deaths are calculated as a function of relative risk (RR), population density (POP), and the baseline mortality rate (BMR). For each model grid cell (*i*, *j*) and health endpoint (*h*), RR is given as

(1) $${\mathrm{RR}}_{i,j,h}=1+\alpha \left[1-\exp\left(-\gamma {\left(C_{i,j}-C_{0}\right)}^{\beta }\right)\right],$$

where *C*_*i,j*_ is the annual mean surface PM_2.5_ concentrations in the grid cell with longitude *i* and latitude *j*; *C*_0_ is the threshold PM_2.5_ concentrations, in our study assumed as 5.8 μg m^−3^ (if *C*_*i,j*_ is equal to or less than 5.8 μg m^−3^, then *RR*_*i,j*_ = 1), by following Burnett et al. (2014); *α*, *γ*, and *β* are model parameters as a result of statistical fitting in determining concentration‐response functions for each health endpoint (Burnett et al., 2014; Morita et al., 2014). For RR in ALRI, we follow Apte et al. (2015) to calculate the RR in each grid box in response to different PM_2.5_ concentrations.

For the impacts of COPD from O_3_, we first follow Jerrett et al. (2009) (hereafter referred to as J2009) to calculate RR, which increases by 4% (95% confidence interval (CI): 1.3%–6.7%) per 10 ppb increases in average daily 1‐hr maximum O_3_ concentrations during April–September. For each grid cell (*i*, *j*), RR is calculated as

(2) $${\mathrm{RR}}_{i,j}=\exp^{\left(\eta Y_{i,j}\right)},$$

where *η* is the log‐linear slope between O_3_ concentrations and RR, with mean value of 0.00392 (95% CI: 0.00129–0.00649). *Y*_*i*,*j*_ is the average daily 1‐hr maximum O_3_ concentration during April–September in the grid cell with longitude *i* and latitude *j*. The minimum and maximum of daily 1‐hr maximum O_3_ concentrations measured during the long‐term cohort studies from J2009 were 33.3 and 104 ppb, respectively. Therefore, we limited the range of *Y*_*i*,*j*_ to 33.3–104 ppb to calculate *RR*_*i*,*j*_ associated with COPD (if *Y*_*i*,*j*_ is less than or equal to 33.3 ppb, *RR*_*i*,*j*_ = 1). If the modeled daily 1‐hr maximum O_3_ concentrations averaged over April–September were greater than 104 ppb, we constrained them as 104 ppb.

In addition, we follow Turner et al. (2016) (hereafter referred to as T2016) for the updated RR from the American Cancer Society Cancer Prevention Study II to calculate O_3_‐induced COPD impacts, with RR shown to increase by 14% (95% CI: 8%–21%) per 10 ppbv increase in annual‐mean daily maximum 8‐hr O_3_ concentrations after adjusting confounding factors of PM_2.5_ and nitrogen dioxides. Compared with J2009, the minimum O_3_ concentration impacts on COPD in T2016 is lower, which is shown to be 26.6 ppb.

The formula for premature mortality in each grid cell (*i*, *j*) and health endpoint (*h*) is demonstrated as

(3) Mi,j,h=POPi,j×BMRi,j,h×RRi,j,h−1RRi,j,h,

where *POP* is the population density in each grid cell and *BMR* is the baseline mortality rate, which is estimated following the GBD2017 study.

Years of life lost (YLL) for each health endpoint (*h*) at each grid cell (*i*, *j*) are calculated as

(4) $${\mathrm{YLL}}_{i,j,h}=M_{i,j,h}\times {\text{MYLL}}_{i,j,h},$$

where *MYLL*_*i,j,h*_ is the mean YLL in each health endpoint attributable to all causes from the GBD2017 study.

**Figure 1:**
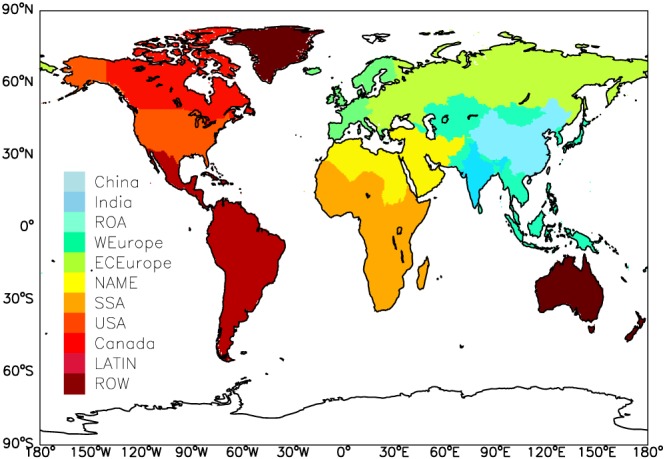
Map of 11 defined regions over continents in our study. ROA = rest of Asia; WEurope = western Europe; ECEurope = eastern and central Europe; NAME = northern Africa and the Middle East; SSA = sub‐Saharan Africa; LATIN = Latin America; ROW = rest of the world.

We estimate the premature mortality and YLL at the horizontal grid resolution of 0.1° × 0.1°. Gridded PM_2.5_ and O_3_ concentrations are regridded from 0.9° × 1.25° to 0.1° × 0.1°. Gridded Population of the World version 4 (GPWv4) is used as the global population density for the year 2015 (Center for International Earth Science Information Network ‐ CIESIN ‐ Columbia University, 2016). We divide the geography of the world into 11 regions in order to compare premature deaths in response to different levels of exposure to PM_2.5_ and O_3_. The regions analyzed are China, India, rest of Asia (ROA), eastern and central Europe (ECEurope), western Europe (WEurope), northern Africa and the Middle East (NAME), sub‐Saharan Africa (SSA), the United States of America (USA), Latin America (LATIN), Canada, and rest of the world (ROW), as shown in Figure 1. For each region, we calculate different BMRs following GBD2017. The health impacts of the five health endpoints are summed to give the total health burden. Uncertainty ranges of premature deaths are from the uncertainty range of RR (2.5%, 50%, and 97.5% CI of RR).

## 3 Results and Discussion

### 3.1 Air Quality Impacts of Global Gasoline and Diesel Emissions

The global gasoline vehicle fleet emits substantial amounts of NMVOCs and CO, with global annual totals of 22,054 and 115,955 kilotons (kt), respectively, for the year 2015 (Table 1), representing ~20% of global total anthropogenic sources of these two pollutants (Table 1). The dominant source regions for NMVOCs and CO are the USA, China, and India (Table 1). In contrast, the global diesel vehicle fleet contributes substantially to BC and NO_x_ emissions, with annual global totals of 952.7 and 22,772 kt for the year 2015, accounting for approximately 14% and 20% of the global total anthropogenic sources, respectively (Table 1).

**Figure 2:**
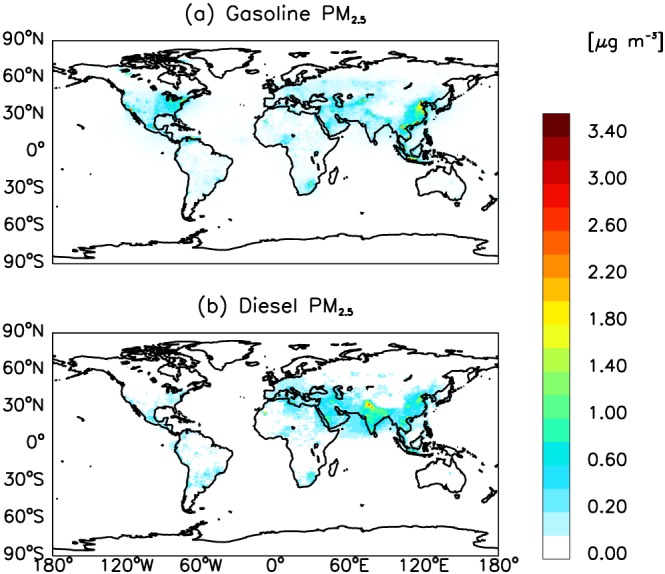
Global annual mean surface PM_2.5_ concentrations for (a) gasoline and (b) diesel vehicle fleet emission sectors.

**Figure 3:**
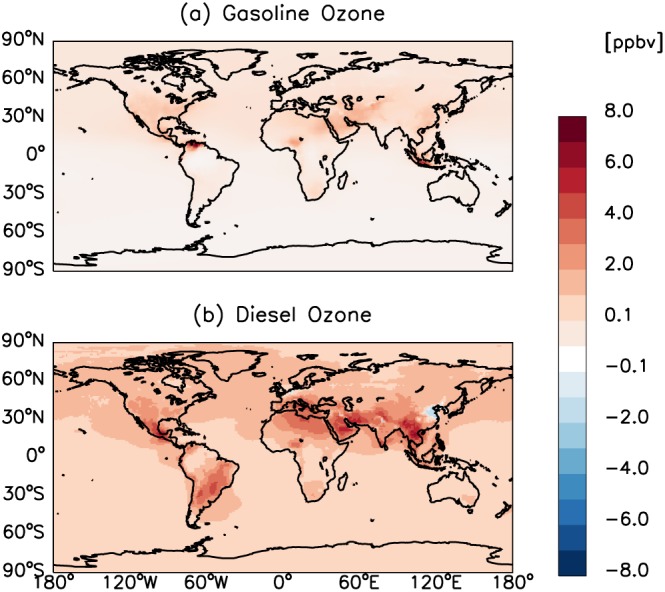
Same as Figure 2, but for surface O_3_.

Global gasoline and diesel emissions lead to increases in annual‐mean surface PM_2.5_ concentrations, by up to 6.0 and 3.0 μg m^−3^, respectively (Figure 2). For gasoline, large increases in surface PM_2.5_ concentrations are found over China, Southeast Asia, and North America (Figure 2a). India, China, and the Middle East are regions associated with substantial increases in surface PM_2.5_ concentrations for the diesel sector (Figure 2b). In terms of surface O_3_, increases in annual‐mean surface O_3_ concentrations attributable to gasoline and diesel sectors are up to 8.5 and 6.7 ppbv, respectively (Figure 3). The former is primarily driven by large amounts of NMVOCs and CO emissions (Table 1), with maximum impacts located over Venezuela and southeastern Asia, followed by the Middle East and USA (Figure 3a). For the latter, model simulations for surface O_3_ concentrations show stronger sensitivity to precursor emissions globally relative to the gasoline sector, which is mostly due to substantial NO_x_ emissions from the diesel sector. Annual‐mean surface O_3_ concentrations for the diesel sector show substantial spatial variability compared with the gasoline sector (Figure 3b). Specifically, the global annual mean surface O_3_ concentration from the diesel sector is ~39% higher than that from the gasoline sector. Interestingly, diesel sector emissions cause decreases in surface O_3_ concentrations over the North China Plain by up to 2.5 ppbv, likely a result of VOC‐limited O_3_ production in this intensely polluted region (Chou et al., 2009; Wang et al., 2017; Xing et al., 2011).

### 3.2 Global Climate Impacts of Gasoline and Diesel Vehicle Fleet Sectors

**Figure 4:**
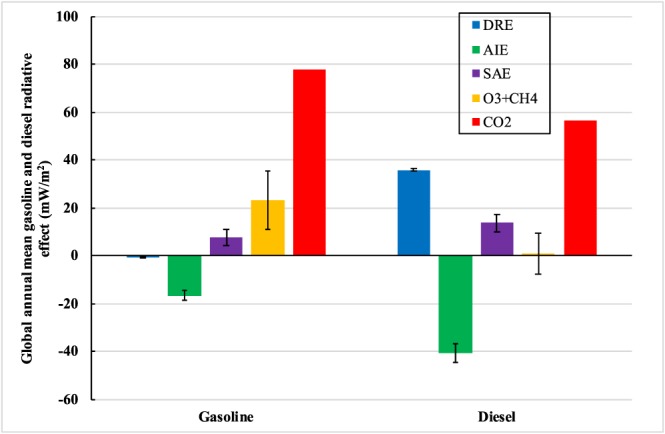
Global annual mean radiative effects for gasoline and diesel emissions, including aerosol direct radiative effect (blue), aerosol indirect effect (green), aerosol surface albedo effect (purple), O_3_ + CH_4_ (orange), and CO_2_ (red). Note that the radiative forcing for CO_2_ is calculated on a 20‐year time horizon. Error bars represent 1 standard deviation.

Figure 4 shows global annual mean radiative effects for gasoline and diesel emissions, including aerosol direct radiative effect (DRE), aerosol indirect effect (AIE), aerosol surface albedo effect (SAE), the combined radiative effects of O_3_ and CH_4_, and the CO_2_ radiative effect on a 20‐year time scale (integrated radiative forcing for the sustained year 2015 emissions). DRE from the diesel sector shows strong positive global warming, with global annual mean radiative effect of +35.8 ± 0.4 (1 σ) mW m^−2^ (Figure 4), which is comparable to the results (+35.4 mW m^−2^) found in Lund et al. (2014). By contrast, the sign of the DRE from gasoline aerosol emissions is negative (cooling) and small: −0.84 ± 0.15 mW m^−2^ (Figure 4). The large difference in the DRE between the gasoline and diesel sectors is because the global annual BC emissions from diesel (952.7 kt year^−1^) are over a factor of six times higher than those from gasoline (148.5 kt year^−1^). Furthermore, CAM5‐Chem assumes strong enhanced BC absorption of solar radiation once coated with soluble species (Huang et al., 2018). The gasoline and diesel sectors are both cooling through the AIE, with global annual mean values of −16.6 ± 2.1 and −40.6 ± 4.0 mW m^−2^, respectively (Figure 4). The cooling effect of the AIE from the gasoline sector is primarily driven by the negative longwave (LW) radiation effect of −22.0 ± 2.0 mW m^−2^, which is partially offset by the positive shortwave (SW) AIE of +5.4 ± 1.8 mW m^−2^ (Table 2). The negative LW and positive SW AIE radiative effects for the gasoline sector can be explained by the strong LW cooling and SW warming effects found over the northern India Ocean (supporting information Figure S1), which is mainly driven by the reductions in deep convective transport of moisture from the surface to the upper troposphere and lower stratosphere (UTLS, Huang et al., 2018), thus leading to decreases in ice cloud fractions over UTLS in this region (Figure S2). This resulted in strong LW cooling and SW warming effects associated with the gasoline sector. In contrast, the SW AIE for the diesel sector (−47.0 ± 5.4 mW m^−2^) dominates the net AIE effect, although a small warming effect from the LW AIE is found (+6.4 ± 2.1 mW m^−2^, Table 2, Figure S3). There are two main reasons contributing to this effect. First, the diesel sector has much higher emissions of BC and POM aerosols, compared with the gasoline sector (Table 1), which increase the cloud droplet number concentrations and cloud liquid water path. As a result, it ends up higher liquid cloud fractions (Figure S4) and thus stronger SW cooling effect for the diesel sector, relative to the gasoline sector. Second, higher BC emissions from the diesel sector considerably enhance the absorption of solar radiation, which strengthens the deep convection by transporting more water vapor from the lower troposphere to UTLS for the formation of ice clouds (Figure S5), leading to globally averaged positive LW radiative effect for the diesel sector (Figure S3). Global annual mean SAEs for the gasoline and diesel sectors are +7.8 ± 3.4 and +13.6 ± 3.6 mW m^−2^, respectively, contributing additional warming. This is mainly due to the deposition of BC aerosols on the surface of snow and sea ice.

**Table 2:** Summary of the Global Radiative Effects of the Gasoline and Diesel Road Vehicle Fleets

Sector	DRE	AIE (SW + LW)	SW AIE	LW AIE	Surface albedo	O_3_ + CH_4_	20‐year CO_2_	100‐year CO_2_
Gasoline	−0.84 ± 0.15	−16.6 ± 2.1	+5.4 ± 1.8	−22.0 ± 2.0	+7.8 ± 3.4	+23.2 ± 12.0	+77.9	+286.8
Diesel	+35.8 ± 0.4	−40.6 ± 4.0	−47.0 ± 5.4	+6.4 ± 2.1	+13.6 ± 3.6	+0.64 ± 8.5	+56.3	+207.2

*Note*. Error bars represent 1 standard deviation. Units: mW m^−2^.

The combined radiative forcing of CH_4_ and O_3_ for the gasoline sector shows substantial overall warming (+23.2 ± 12.0 mW m^−2^) through both CH_4_ and O_3_ driven by the impacts of CO and NMVOCs (Figure 4 and Table 2). For diesel, the net forcing is almost negligible (+0.64 ± 8.5 mW m^−2^) because NO_x_‐driven O_3_ warming is offset by the NO_x_‐driven CH_4_ cooling. In comparison, Lund et al. (2014) found that the combined radiative forcing of CH_4_ and O_3_ in the diesel sector for the year 2010 was of opposite sign, −9 mW m^−2^. We calculate the short‐term (20‐year time scale) and long‐term (100‐year time scale) CO_2_ radiative forcing for the gasoline and diesel sectors using IPCC climate policy metrics (Myhre et al., 2013). The radiative forcing of CO_2_ is calculated as a function of CO_2_ absolute global warming potential (W m^−2^ year (kg CO_2_)^−1^) and annual emission fluxes (kg year^−1^). Absolute global warming potentials for CO_2_ over 20‐year and 100‐year time horizons are 2.49 × 10^−14^ and 9.17 × 10^−14^ W m^−2^ year (kg CO_2_)^−1^ (Myhre et al., 2013), respectively. CO_2_ emissions from the gasoline and diesel sectors for the year 2015 are 3.13 × 10^9^ and 2.26 × 10^9^ kg year^−1^. Therefore, CO_2_ radiative forcings on the 20‐year time scale for year 2015 emissions from the global gasoline and diesel vehicle fleets are +77.9 and +56.3 mW m^−2^, respectively. For comparison, on the 100‐year time scale, the CO_2_ radiative forcing is +286.8 (gasoline) and +207.2 mW m^−2^ (diesel), completely dwarfing SLCF impacts.

Thus, the net total radiative effects of the global gasoline and diesel on‐road vehicle fleets for year 2015 emissions on the 20‐year time scale (including CO_2_ and SLCFs) are +91.4 (gasoline) and +65.7 mW m^−2^ (diesel). The combined net radiative forcing for both fuel types is +157.1 mW m^−2^, which is consistent with previous studies (Unger et al., 2009, 2010). The fractional contributions of SLCF forcings for the gasoline and diesel sectors relative to the total radiative forcing on a 20‐year time scale are similar: approximately 14.9% and 14.4%, respectively.

### 3.3 PM_2.5_‐ and O_3_‐Induced Premature Deaths of Gasoline and Diesel Emissions

Global PM_2.5_‐ and O_3_‐induced premature deaths exceed four million people for five health endpoints calculated in our study, which is consistent with previous studies (Anenberg et al., 2017; Lelieveld et al., 2015, 2019), with global total YLL approaching 80 million years (Table 3). As mentioned earlier, we have followed J2009 and T2016 methods to quantify O_3_‐induced COPD separately. When using J2009, global total PM_2.5_‐ and O_3_‐induced premature deaths associated with the gasoline and diesel sectors for the year 2015 are 86,400 (95% CI: 41,000–126,000) and 89,100 (95% CI: 40,600–128,900), respectively, accounting for about 2.2% and 2.3% of the baseline. The fractional contributions of PM_2.5_‐ versus O_3_‐induced premature deaths relative to the totals are approximately 75.6% and 24.4% for gasoline and 52.8% and 47.2% for diesel (Table 3). In terms of YLL, annual total YLL associated with PM_2.5_‐ and O_3_‐induced premature deaths for the gasoline and diesel sectors are quite comparable: 1.56 (95% CI: 0.80–2.25) and 1.66 (95% CI: 0.85–2.41) million years, respectively.

**Table 3:** Global PM_2.5_‐ and O_3_‐Induced Premature Deaths

Cases	Species	Premature deaths (×1,000 persons)	YLL (×10^6^ year)
CONTROL	PM_2.5_	3,581 (1,854, 4,865)	75.0 (42.3, 99.9)
	Ozone^a^	361.2 (125.8, 566.5)	5.58 (1.94, 8.76)
	Ozone^b^	1,042.1 (677.6, 1,365.8)	16.4 (10.7, 21.5)
Gasoline (CONTROL – Gasoline removed)	PM_2.5_	65.3 (33.4, 94.9)	1.24 (0.68, 1.77)
	Ozone^a^	21.1 (7.69, 31.7)	0.32 (0.12, 0.48)
	Ozone^b^	49.7 (35.6, 58.7)	0.76 (0.54, 0.90)
Diesel (CONTROL – Diesel removed)	PM_2.5_	46.9 (25.4, 67.7)	1.00 (0.61, 1.41)
	Ozone^a^	42.2 (15.2, 64.2)	0.66 (0.24, 1.00)
	Ozone^b^	75.2 (53.1, 89.8)	1.20 (0.85, 1.44)

*Note*. Parenthesis is the uncertainty range, which is solely based on the uncertainty of relative risk. YLL = years of life lost.

a Method follows J2009.

b Method follows T2016.

**Figure 5:**
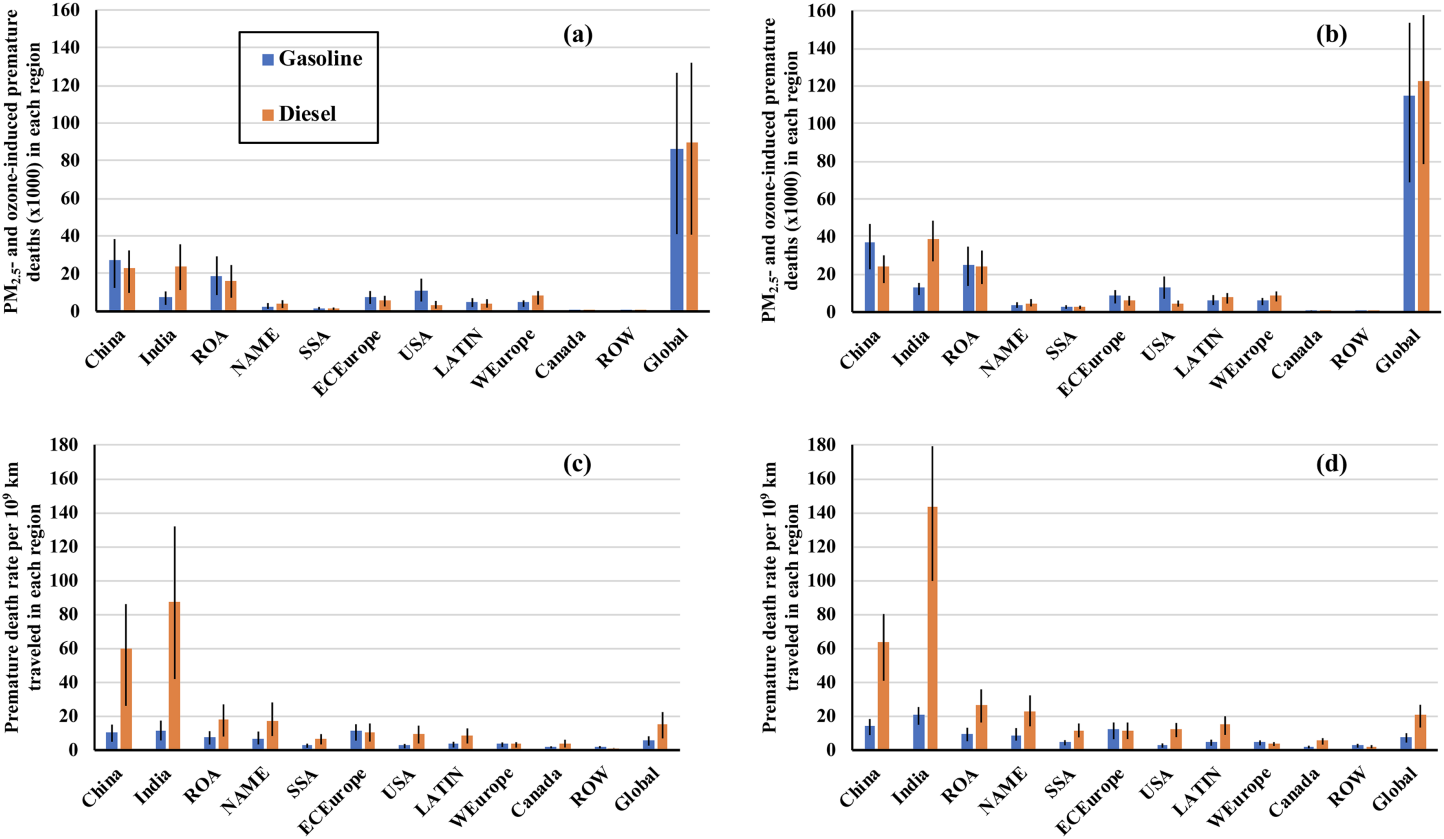
PM_2.5_‐ and O_3_‐induced annual‐mean premature deaths (a, b) and premature death rate per 10^9^ km traveled (c, d) from emissions of gasoline (blue) and diesel (orange) for 11 different regions, with O_3_ impacts on COPD following J2009 shown in (a, c) and T2016 shown in (b, d), respectively. The regional premature death rate is calculated as the regional total premature deaths divided by the total annual regional distances traveled for each fuel type. Error bars represent 95% confidence intervals, which is solely based on the uncertainty range of relative risk.

With the updated RR associated with O_3_ impacts on COPD following T2016, global annual O_3_‐induced premature deaths increase by 136% and 78% for the gasoline and diesel sectors relative to the case using J2009, which results in increases of 33% and 37% for the annual total combined PM_2.5_‐ and O_3_‐induced premature deaths relative to J2009 for the gasoline and diesel sectors, respectively. YLL increases by 0.44 and 0.54 million years for the gasoline and diesel sectors when using updated RR associated with O_3_‐induced COPD, compared with J2009. The combined PM_2.5_‐ and O_3_‐induced premature deaths (YLL) from the diesel sector for the year 2015 with T2016 are ~6% (10%) higher than those from global gasoline impacts (Table 3). Gasoline emissions in Asia (including China, India, and other Asian regions), USA, and eastern and central Europe are the largest contributors (Table 4), with combined premature deaths from PM_2.5_ and O_3_ accounting for ~83% relative to the global total. Interestingly, global on‐road diesel fuel usage is mainly concentrated in Asia and Europe, with premature death contributions of 71% and 12.5% relative to the global sum. In particular, premature deaths from the diesel sector in India and western Europe are both higher than the impacts from the gasoline sector due to the high penetration of diesel vehicles in these two regions (Figures 5a and 5b). We acknowledge that the relatively coarse spatial resolution of our model simulation (0.9° latitude × 1.25° longitude) is a major source of uncertainty associated with the quantification of premature deaths for the gasoline and diesel sectors. However, the spatial resolution of the model that we apply in our study is typically available for the current generation of global chemistry‐climate models (Turnock et al., 2020).

**Table 4:** PM_2.5_‐ and O_3_‐Induced Premature Deaths (×1,000) for Each Region and Case

Region		CONTROL	Gasoline	Diesel
China	PM_2.5_	1,262 (575.5, 1,782)	18.7 (9.42, 26.5)	10.0 (5.16, 14.0)
	Ozone^a^	156.4 (55.2, 242.4)	8.09 (3.04, 11.8)	12.5 (4.64, 18.3)
	Ozone^b^	376.0 (246.7, 487.9)	17.9 (13.2, 20.2)	14.0 (10.2, 16.1)
India	PM_2.5_	826.9 (455.3, 1,191)	3.47 (2.16, 5.13)	11.1 (6.83, 16.6)
	Ozone^a^	96.7 (33.5, 152.5)	3.62 (1.31, 5.45)	12.5 (4.48, 19.0)
	Ozone^b^	338.8 (223.0, 438.6)	9.23 (6.90, 10.3)	27.6 (20.1, 31.8)
ROA	PM_2.5_	599.4 (302.8, 819.3)	15.2 (7.32, 23.3)	10.3 (5.17, 15.5)
	Ozone^a^	35.9 (12.4, 56.9)	3.83 (1.36, 5.88)	5.87 (2.08, 9.07)
	Ozone^b^	122.6 (78.9, 162.5)	9.39 (6.51, 11.4)	14.0 (9.68, 17.1)
NAME	PM_2.5_	238.5 (137.1, 329.6)	1.92 (1.07, 3.24)	2.32 (1.28, 3.91)
	Ozone^a^	9.72 (3.36, 15.4)	0.74 (0.27, 1.13)	1.34 (0.48, 2.03)
	Ozone^b^	27.1 (17.4, 36.0)	1.64 (1.16, 1.96)	2.42 (1.69, 2.90)
SSA	PM_2.5_	205.2 (123.3, 264.8)	1.09 (0.70, 1.51)	0.85 (0.55, 1.18)
	Ozone^a^	3.60 (1.22, 5.83)	0.50 (0.17, 0.81)	0.54 (0.18, 0.86)
	Ozone^b^	17.3 (10.6, 23.9)	1.59 (1.02, 2.10)	1.68 (1.08, 2.22)
ECEurope	PM_2.5_	198.1 (123.9, 204.4)	7.41 (3.79, 10.1)	4.28 (2.16, 6.08)
	Ozone^a^	8.56 (2.92, 13.7)	0.47 (0.16, 0.72)	1.38 (0.49, 2.15)
	Ozone^b^	21.8 (13.6, 29.8)	1.16 (0.77, 1.49)	1.91 (1.26, 2.44)
USA	PM_2.5_	101.5 (54.6, 126.0)	9.42 (4.58, 14.4)	1.43 (0.69, 2.24)
	Ozone^a^	22.3 (7.73, 35.1)	1.93 (0.70, 2.90)	2.17 (0.79, 3.26)
	Ozone^b^	54.3 (34.6, 72.4)	3.62 (2.51, 4.39)	3.18 (2.21, 3.85)
LATIN	PM_2.5_	74.4 (41.7, 86.2)	3.92 (2.01, 5.59)	2.64 (1.36, 3.69)
	Ozone^a^	6.07 (2.05, 9.78)	0.89 (0.31, 1.42)	1.73 (0.59, 2.76)
	Ozone^b^	25.5 (15.9, 34.8)	2.59 (1.69, 3.39)	4.87 (3.15, 6.39)
WEurope	PM_2.5_	57.7 (34.7, 59.0)	3.81 (2.07, 4.46)	3.91 (2.15, 4.39)
	Ozone^a^	20.5 (7.01, 32.7)	0.91 (0.32, 1.41)	4.16 (1.47, 6.43)
	Ozone^b^	54.3 (34.1, 73.8)	2.35 (1.58, 2.97)	5.22 (3.50, 6.56)
Canada	PM_2.5_	5.95 (4.17, 6.59)	0.35 (0.16, 0.55)	0.09 (0.04, 0.13)
	Ozone^a^	1.24 (0.42, 1.97)	0.11 (0.04, 0.17)	0.13 (0.05, 0.21)
	Ozone^b^	3.42 (2.15, 4.66)	0.22 (0.15, 0.28)	0.21 (0.14, 0.26)
ROW	PM_2.5_	3.39 (1.94, 3.47)	0.08 (0.04, 0.11)	0.04 (0.02, 0.06)
	Ozone^a^	0.14 (0.05, 0.22)	0.02 (0.006, 0.03)	0.03 (0.01, 0.05)
	Ozone^b^	1.03 (0.62, 1.45)	0.08 (0.05, 0.11)	0.14 (0.08, 0.19)

*Note*. Parenthesis is the uncertainty range, which is solely based on the uncertainty of relative risk.

a Method follows J2009.

b Method follows T2016.

### 3.4 Normalization of Health Effects by Vehicle Distance Traveled

Global total vehicle distance traveled for the gasoline sector in 2015 was about 1.55 × 10^13^ km, which is about 2.6 times higher than that from the diesel sector (Table 5). Gasoline vehicles in the USA account for the largest contribution (31.2%) relative to the global total distances of gasoline vehicles traveled, followed by ROA (17.0%) and China (16.5%). Distance traveled by diesel vehicles from western Europe accounts for approximately 39.9% of the global total distances traveled by diesel vehicles, followed by ROA (15.5%) and ECEurope (8.9%). Thus, half of the annual total distance traveled by diesel vehicles was in Europe alone.

**Table 5:** Vehicle Distance Traveled and Premature Death Rate per 10^9^ km in Each Region for Each Fuel Type

Region	Gasoline vehicledistancetraveled(10^9^ km)	Dieselvehicle distancetraveled(10^9^ km)	Gasoline prematuremortality rate (10^−9^deaths km^−1^)^a^	Gasoline prematuremortality rate(10^−9^ deaths km^−1^)^b^	Diesel premature mortality rate (10^−9^ deaths km^−1^)^a^	Diesel premature mortality rate (10^−9^ deaths km^−1^)^b^
China	2,556.7	375.4	10.5 (4.87, 15.0)	14.3 (8.85, 18.3)	59.9 (26.1, 86.0)	63.9 (40.9, 80.2)
India	608.0	270.1	11.7 (5.71, 17.4)	20.9 (14.9, 25.4)	87.4 (41.9, 131.8)	143.3 (99.7, 179.2)
ROA	2,633.1	910.7	7.23 (3.30, 11.1)	9.34 (5.25, 13.2)	17.8 (7.96, 27.0)	26.7 (16.3, 35.8)
NAME	401.6	211.6	6.62 (3.34, 10.9)	8.87 (5.55, 12.9)	17.3 (8.32, 28.1)	22.4 (14.0, 32.2)
SSA	622.7	217.3	2.55 (1.40, 3.73)	4.30 (2.76, 5.80)	6.40 (3.36, 9.39)	11.6 (7.50, 15.6)
ECEurope	711.8	525.1	11.1 (5.55, 15.2)	12.0 (6.41, 16.3)	10.8 (5.05, 15.7)	11.8 (6.51, 16.2)
USA	4,838.5	381.5	2.35 (1.09, 3.58)	2.70 (1.47, 3.88)	9.44 (3.88, 14.4)	12.1 (7.60, 16.0)
LATIN	1,454.9	505.3	3.31 (1.59, 4.82)	4.47 (2.54, 6.17)	8.65 (3.86, 12.8)	14.9 (8.93, 19.9)
WEurope	1,310.2	2,353.6	3.60 (1.82, 4.48)	4.70 (2.79, 5.67)	3.43 (1.54, 4.60)	3.88 (2.40, 4.65)
Canada	321.5	55.8	1.43 (0.62, 2.24)	1.77 (0.96, 2.58)	3.94 (1.61, 6.10)	5.38 (3.23, 6.99)
ROW	60.5	86.6	1.65 (0.76, 2.31)	2.64 (1.49, 3.64)	0.81 (0.35, 1.27)	2.08 (1.15, 2.89)
Global	1,5519.4	5892.9	5.57 (2.64, 8.16)	7.42 (4.44, 9.89)	15.2 (6.90, 21.4)	20.7 (13.3, 26.7)

*Note*. Parenthesis is the uncertainty range.

a Method follows J2009.

b Method follows T2016.

We normalize the regional total premature deaths from PM_2.5_ and O_3_ by vehicle distance traveled for each fuel type in each region (Table 5) to provide a premature death rate metric in units of deaths per kilometer. Following J2009, global mean diesel premature death rate is 15.2 × 10^−9^ deaths km^−1^, which is ~2.7 times higher than that from the gasoline sector (Figure 5c). For the gasoline sector, regional premature death rates vary by a factor of eight, ranging from 1.43 × 10^−9^ deaths km^−1^ in Canada to 1.17 × 10^−8^ deaths km^−1^ in India. In contrast, substantial regional variabilities of premature death rates associated with the diesel sector are found, the variability of which can be up to two orders of magnitude (Figure 5c), with the lowest in ROW (0.81 × 10^−9^ deaths km^−1^) and the highest in India (8.7 × 10^−8^ deaths km^−1^). The highest regional premature death rates for both the gasoline and diesel sectors are found in India due to a combination of dense regional population for exposure to PM_2.5_ and O_3_, relatively low annual total vehicle distance traveled as well as higher tailpipe emissions associated with adopted lower fuel quality for each fuel type in India (Miller et al., 2017). When using T2016 to calculate O_3_ impacts on COPD, updated global average premature death rates for the diesel and gasoline sectors increase by 36.2% and 33.9%, respectively, compared with the case using J2009. This result is because global O_3_‐induced premature deaths for COPD using T2016 are about two times higher than the case using J2009 (section 3.3), leading to increases in all regional premature death rates for both sectors, especially for the diesel sector where O_3_‐induced premature deaths are quite comparable to PM_2.5_‐induced premature deaths using J2009 (Table 3). Therefore, regional variability of total PM_2.5_‐ and O_3_‐induced premature death rates for the gasoline and diesel sectors between the J2009 and the T2016 case is quite low (Figures 5c and 5d). We acknowledge that we don't quantify the uncertainties associated with emission inventories and vehicle distance traveled for gasoline and diesel sectors in this study, which merits further investigations in the future.

### 3.5 Comparisons to Previous Studies

In this study, the combined PM_2.5_‐ and O_3_‐induced premature deaths for the year 2015 from the on‐road gasoline and diesel subsectors are up to 237,000 (95% CI: 147,000–311,000), which is comparable to the recent study by Anenberg et al. (2019), who reported that on‐road diesel and nondiesel emissions caused about 246,000 premature deaths associated with PM_2.5_ and O_3_. Differences in the results across studies are by application of different year 2015 emission inventories, the IIASA GAINS ECLIPSE V5a inventory is used here versus the International Council on Clean Transportation (Miller & Jin, 2018). Moreover, Anenberg et al. (2019) assess “nondiesel” emissions as gasoline but also other fuels such as liquefied petroleum gas and compressed natural gas. Several other studies have quantified health effects from the global transportation sector. For example, Chambliss et al. (2014) found that emissions from the surface transportation sector resulted in approximately 242,000 premature deaths attributable to surface PM_2.5_ pollution for the year 2005. Their estimate includes on‐road and off‐road transportation emissions for all fuel sources. Silva et al. (2016) estimated that PM_2.5_‐ and O_3_‐induced premature deaths from the entire global land transportation sector in year 2010 were about 376,000 for the year 2005, about 59% higher than here for the on‐road diesel and gasoline subsectors in year 2015. A likely source of the discrepancy aside from the sector definition is the different baseline mortality rates associated with each disease for different years (GBD2017 for the year 2015 in our study versus GBD2010 for the year 2005 in Silva et al., 2016). Furthermore, the model applied in this study, CAM5‐Chem, does not explicitly account for nitrate aerosol formation, which may lead to underestimated annual‐mean total PM_2.5_ concentrations and thus lower PM_2.5_‐induced premature deaths. Global averaged percentage of surface nitrate aerosol mass relative to the total PM mass based on ensemble mean of observations is about 9.9% (Zhang et al., 2007). By scaling up the gridded surface PM_2.5_ concentrations by 9.9% for all model simulations to account for the contributions of nitrate aerosols, we find that, on average, there are additional PM_2.5_‐induced premature deaths of 5,800 and 4,600 for the gasoline and diesel sectors, respectively. Lelieveld et al. (2015) reported that the global land transportation sector caused ~164,000 deaths due to PM_2.5_ and O_3_ for the year 2010. The lower estimate is explained by the substantially lower estimates of mortality associated with O_3_ and the higher counterfactual threshold values for PM_2.5_ concentrations (e.g., 7.5 μg m^−3^) in Lelieveld et al. (2015), compared with this study and other recent work.

## 4 Conclusion

In this study, we employed the NCAR CESM CAM5‐Chem model to investigate the impacts of gasoline and diesel emissions on air quality, climate, and human health. The IIASA GAINS ECLIPSE V5a emissions inventory is used as the baseline anthropogenic emissions inventory. For the global gasoline on‐road sector, NMVOCs and CO emissions account for approximately 20% of the total anthropogenic emissions. The global diesel on‐road vehicle fleet contributes substantially to global BC and NO_x_ emissions, which are about 14% and 20% of the total anthropogenic emissions. As a result, global gasoline and diesel emissions lead to regional increases in annual mean surface PM_2.5_ concentrations by up to 6.0 and 3.0 μg m^−3^, and surface O_3_ concentrations by up to 8.5 and 6 ppbv, respectively. We find substantial scope for mitigation of on‐road diesel and gasoline emissions to improve human health and contribute to international climate goals.

Net radiative effects of SLCFs, including aerosols, O_3_, and CH_4_, from the gasoline and diesel sectors are +13.6 and +9.4 mW m^−2^, respectively. Specifically, global annual mean net aerosol radiative effects for the gasoline and diesel sectors are −9.6 ± 2.0 and +8.8 ± 5.8 mW m^−2^. Global annual mean DRE for the gasoline and diesel sectors have different signs (−0.84 ± 0.15 versus +35.8 ± 0.4 mW m^−2^), whereas the global annual mean AIE are both cooling (−16.6 ± 2.1 and −40.6 ± 4.0 mW m^−2^). However, the underlying aerosol‐cloud microphysical drivers are different, with net cooling AIE for gasoline and diesel sectors dominated by the LW AIE (−22 ± 2.0 mW m^−2^) and the SW AIE (−47.0 ± 5.4 mW m^−2^), respectively. Due to the deposition and absorption of solar radiation of BC on snow and sea ice, the net effect of SAE for both sectors are warming, with global annual averages of +7.8 ± 3.4 and +13.6 ± 3.6 mW m^−2^. The combined radiative forcing of CH_4_ and O_3_ for the gasoline sector is warming (+23.2 ± 12.0 mW m^−2^), primarily driven by the impacts of CO and NMVOCs. In contrast, the net forcing of CH_4_ and O_3_ for the diesel sector is almost negligible because the NO_x_‐driven O_3_ warming is offset by the NO_x_‐driven CH_4_ cooling. In comparison, the CO_2_ radiative forcing on the 20‐year timescale for year 2015 emissions are +77.9 (gasoline) and +56.3 mW m^−2^ (diesel). SLCFs contribute similar magnitudes, 14.9% (gasoline) and 14.4% (diesel), to the net global climate impact for year 2015 emissions on a 20‐year timescale.

In terms of health effects associated with the gasoline and diesel sectors, global annual PM_2.5_‐ and O_3_‐induced premature deaths are 86,400 (95% CI: 41,000–126,000) for gasoline and 89,100 (95% CI: 40,600–128,900) for diesel, with the corresponding YLL of 1.56 (95% CI: 0.80–2.25) and 1.66 (95% CI: 0.85–2.41) million years for the gasoline and diesel sectors, respectively. Here the O_3_‐induced COPD impacts follow J2009. When updated to T2016 for the O_3_‐induced COPD, global annual total PM_2.5_‐ and O_3_‐induced premature deaths increase by 33% and 37% for the gasoline and diesel sectors, compared with the case using J2009. Consequently, YLL increases by 0.44 and 0.54 million years for gasoline and diesel sectors.

Diesel premature death rates (total premature deaths normalized by annual total distance traveled for each fuel type in each region, in units of deaths per km) show substantial regional variability, with the diesel regional premature death rate in India up to two orders of magnitude higher than that in other regions. This is primarily due to higher premature deaths and relatively lower distance traveled in India for the diesel sector, compared with other regions. For the gasoline sector, regional premature death rates vary by about a factor of eight. India is the region that shows the highest premature death rate for both the gasoline and diesel sectors. Updated global‐average premature death rates for the diesel and gasoline sectors using T2016 increase by 36.2% and 33.9%, respectively, compared with the case using J2009 associated with O_3_‐induced COPD. Our study is the first to report this novel metric to highlight regions with high potential to achieve co‐benefits of air quality and public health associated with gasoline and diesel sectors. In addition, our study is also one of the first studies to quantify the integrated climate and health effects from the on‐road transportation sectors using a consistent model framework. In terms of climatic impacts, our study is also the first to employ the online aerosol‐cloud interactions using CAM5‐Chem to estimate the first and second aerosol indirect effects for aerosol emissions from gasoline and diesel sectors.

## Conflict of interest

The authors declare no conflicts of interest relevant to this study.

## Supporting information

Supporting Information S1

## References

[gh2147-bib-0001] Amann, M. , Bertok, I. , Borken‐Kleefeld, J. , Cofala, J. , Heyes, C. , Höglund‐Isaksson, L. , Klimont, Z. , Nguyen, B. , Posch, M. , Rafaj, P. , Sandler, R. , Schöpp, W. , Wagner, F. , & Winiwarter, W. (2011). Cost‐effective control of air quality and greenhouse gases in Europe: Modeling and policy applications. Environmental Modelling and Software, 26(12), 1489–1501. 10.1016/j.envsoft.2011.07.012

[gh2147-bib-0002] Amann, M. , Klimont, Z. , & Wagner, F. (2013). Regional and global emissions of air pollutants: Recent trends and future scenarios. Annual Review of Environment and Resources, 38(1), 31–55. 10.1146/annurev-environ-052912-173303

[gh2147-bib-0003] Anenberg, S. , Miller, J. , Henze, D. , & Minjares, R. (2019). A global snapshot of the air pollution‐related health impacts of transportation sector emissions in 2010 and 2015. Retrieved from http://www.theicct.org

[gh2147-bib-0004] Anenberg, S. C. , Miller, J. , Minjares, R. , du, L. , Henze, D. K. , Lacey, F. , Malley, C. S. , Emberson, L. , Franco, V. , Klimont, Z. , & Heyes, C. (2017). Impacts and mitigation of excess diesel‐related NOx emissions in 11 major vehicle markets. Nature, 545(7655), 467–471. 10.1038/nature22086 28505629

[gh2147-bib-0005] Apte, J. S. , Marshall, J. D. , Cohen, A. J. , & Brauer, M. (2015). Addressing global mortality from ambient PM_2.5_ . Environmental Science and Technology, 49(13), 8057–8066. 10.1021/acs.est.5b01236 26077815

[gh2147-bib-0006] Balkanski, Y. , Myhre, G. , Gauss, M. , Rädel, G. , Highwood, E. J. , & Shine, K. P. (2010). Direct radiative effect of aerosols emitted by transport: From road, shipping and aviation. Atmospheric Chemistry and Physics, 10(10), 4477–4489. 10.5194/acp-10-4477-2010

[gh2147-bib-0007] Ban‐Weiss, G. A. , McLaughlin, J. P. , Harley, R. A. , Lunden, M. M. , Kirchstetter, T. W. , Kean, A. J. , Strawa, A. W. , Stevenson, E. D. , & Kendall, G. R. (2008). Long‐term changes in emissions of nitrogen oxides and particulate matter from on‐road gasoline and diesel vehicles. Atmospheric Environment, 42(2), 220–232. 10.1016/j.atmosenv.2007.09.049

[gh2147-bib-0008] Barrett, S. R. H. , Speth, R. L. , Eastham, S. D. , Dedoussi, I. C. , Ashok, A. , Malina, R. , & Keith, D. W. (2015). Impact of the Volkswagen emissions control defeat device on US public health Impact of the Volkswagen emissions control defeat device on US public health. Environmental Research Letters, 10, 114005.

[gh2147-bib-0009] Burnett, R. T. , Pope, C. A. III , Ezzati, M. , Olives, C. , Lim, S. S. , Mehta, S. , Shin, H. H. , Singh, G. , Hubbell, B. , Brauer, M. , Anderson, H. R. , Smith, K. R. , Balmes, J. R. , Bruce, N. G. , Kan, H. , Laden, F. , Prüss‐Ustün, A. , Turner, M. C. , Gapstur, S. M. , Diver, W. R. , & Cohen, A. (2014). An integrated risk function for estimating the global burden of disease attributable to ambient fine particulate matter exposure. Environmental Health Perspectives, 122(4), 397–403. 10.1289/ehp.1307049 24518036 PMC3984213

[gh2147-bib-0010] Center for International Earth Science Information Network ‐ CIESIN ‐ Columbia University . (2016). Gridded Population of the World, Version 4 (GPWv4): Population density. Palisades, NY. 10.7927/H4NP22DQ

[gh2147-bib-0011] Chambliss, S. E. , Silva, R. , West, J. J. , Zeinali, M. , & Minjares, R. (2014). Estimating source‐attributable health impacts of ambient fine particulate matter exposure: Global premature mortality from surface transportation emissions in 2005. Environmental Research Letters, 9(10). 10.1088/1748-9326/9/10/104009

[gh2147-bib-0012] Chou, C. C. K. , Tsai, C. Y. , Shiu, C. J. , Liu, S. C. , & Zhu, T. (2009). Measurement of NOy during campaign of air quality research in Beijing 2006 (CAREBeijing‐2006): Implications for the ozone production efficiency of NOx. Journal of Geophysical Research, 114, D00G01 10.1029/2008JD010446

[gh2147-bib-0013] Collins, W. J. , Fry, M. M. , Yu, H. , Fuglestvedt, J. S. , Shindell, D. T. , & West, J. J. (2013). Global and regional temperature‐change potentials for near‐term climate forcers. Atmospheric Chemistry and Physics, 13(5), 2471–2485. 10.5194/acp-13-2471-2013

[gh2147-bib-0014] Emmons, L. K. , Walters, S. , Hess, P. G. , Lamarque, J. F. , Pfister, G. G. , Fillmore, D. , Granier, C. , Guenther, A. , Kinnison, D. , Laepple, T. , Orlando, J. , Tie, X. , Tyndall, G. , Wiedinmyer, C. , Baughcum, S. L. , & Kloster, S. (2010). Description and evaluation of the Model for Ozone and Related chemical Tracers, version 4 (MOZART‐4). Geoscientific Model Development, 3(1), 43–67. 10.5194/gmd-3-43-2010

[gh2147-bib-0015] Fuglestvedt, J. S. , Shine, K. P. , Berntsen, T. , Cook, J. , Lee, D. S. , Stenke, A. , Skeie, R. B. , Velders, G. J. M. , & Waitz, I. A. (2010). Transport impacts on atmosphere and climate: Metrics. Atmospheric Environment, 44(37), 4648–4677. 10.1016/j.atmosenv.2009.04.044 PMC7110594 32288556

[gh2147-bib-0016] Fuglestvedt, J. , Berntsen, T. , Myhre, G. , Rypdal, K. , & Skeie, R. B. (2008). Climate forcing from the transport sectors. Proceedings of the National Academy of Sciences, 105(2), 454–458. 10.1073/pnas.0702958104 PMC2206557 18180450

[gh2147-bib-0017] Ghan, S. J. (2013). Technical note: Estimating aerosol effects on cloud radiative forcing. Atmospheric Chemistry and Physics, 13(19), 9971–9974. 10.5194/acp-13-9971-2013

[gh2147-bib-0018] Ghan, S. J. , Liu, X. , Easter, R. C. , Zaveri, R. , Rasch, P. J. , Yoon, J. H. , & Eaton, B. (2012). Toward a minimal representation of aerosols in climate models: Comparative decomposition of aerosol direct, semidirect, and indirect radiative forcing. Journal of Climate, 25(19), 6461–6476. 10.1175/JCLI-D-11-00650.1

[gh2147-bib-0019] Granier, C. , & Brasseur, G. P. (2003). The impact of road traffic on global tropospheric ozone. Geophysical Research Letters, 30(2), 1086 10.1029/2002GL015972

[gh2147-bib-0020] Hill, J. , Polasky, S. , Nelson, E. , Tilman, D. , Huo, H. , Ludwig, L. , Neumann, J. , Zheng, H. , & Bonta, D. (2009). Climate change and health costs of air emissions from biofuels and gasoline. Proceedings of the National Academy of Sciences, 106(6), 2077–2082. 10.1073/pnas.0812835106 PMC2634804 19188587

[gh2147-bib-0021] Holland, S. P. , Mansur, E. T. , Muller, N. Z. , & Yates, A. J. (2016). Damages and expected deaths due to excess NO_x_ emissions from 2009 to 2015 Volkswagen diesel vehicles. Environmental Science and Technology, 50(3), 1111–1117. 10.1021/acs.est.5b05190 26720281

[gh2147-bib-0022] Hoor, P. , Borken‐Kleefeld, J. , Caro, D. , Dessens, O. , Endresen, O. , Gauss, M. , Grewe, V. , Hauglustaine, D. , Isaksen, I. S. A. , Jöckel, P. , Lelieveld, J. , Myhre, G. , Meijer, E. , Olivie, D. , Prather, M. , Schnadt Poberaj, C. , Shine, K. P. , Staehelin, J. , Tang, Q. , van Aardenne, J. , van Velthoven, P. , & Sausen, R. (2009). The impact of traffic emissions on atmospheric ozone and OH: Results from QUANTIFY. Atmospheric Chemistry and Physics, 9(9), 3113–3136. 10.5194/acp-9-3113-2009

[gh2147-bib-0023] Hou, L. , Zhang, K. , Luthin, M. , & Baccarelli, A. (2016). Public health impact and economic costs of Volkswagen's lack of compliance with the United States' Emission Standards. International Journal of Environmental Research and Public Health, 13(9), 891 10.3390/ijerph13090891 PMC5036724 27618076

[gh2147-bib-0024] Huang, Y. , Unger, N. , Storelvmo, T. , Harper, K. , Zheng, Y. , & Heyes, C. (2018). Global radiative effects of solid fuel cookstove aerosol emissions. Atmospheric Chemistry and Physics, 18(8), 5219–5233. 10.5194/acp-18-5219-2018

[gh2147-bib-0025] Huang, Y. , Wu, S. , Kramer, L. J. , Helmig, D. , & Honrath, R. E. (2017). Tropospheric ozone and its precursors at Summit, Greenland: Comparison between observations and model simulations. Atmospheric Chemistry and Physics, 17, 14661–14674. 10.5194/acp-2017-14661-2017

[gh2147-bib-0026] Iacono, M. J. , Delamere, J. S. , Mlawer, E. J. , Shephard, M. W. , Clough, S. A. , & Collins, W. D. (2008). Radiative forcing by long‐lived greenhouse gases: Calculations with the AER radiative transfer models. Journal of Geophysical Research, 113, D13103 10.1029/2008JD009944

[gh2147-bib-0027] Jacobson, M. Z. (2007). Effects of ethanol (E85) versus gasoline vehicles on cancer and mortality in the United States. Environmental Science and Technology, 41(11), 4150–4157. 10.1021/es062085v 17612204

[gh2147-bib-0028] Jerrett, M. , Burnett, R. T. , Pope, C. A. III , Ito, K. , Thurston, G. , Krewski, D. , Shi, Y. , Calle, E. , & Thun, M. (2009). Long‐term ozone exposure and mortality. New England Journal of Medicine, 360(11), 1085–1095. 10.1056/NEJMoa0803894 19279340 PMC4105969

[gh2147-bib-0029] Kanakidou, M. , Seinfeld, J. H. , Pandis, S. N. , Barnes, I. , Dentener, F. J. , Facchini, M. C. , van Dingenen, R. , Ervens, B. , Nenes, A. , Nielsen, C. J. , Swietlicki, E. , Putaud, J. P. , Balkanski, Y. , Fuzzi, S. , Horth, J. , Moortgat, G. K. , Winterhalter, R. , Myhre, C. E. L. , Tsigaridis, K. , Vignati, E. , Stephanou, E. G. , & Wilson, J. (2005). Organic aerosol and global climate modelling: A review. Section Title: Air Pollution and Industrial Hygiene, 5(4), 1053–1123. 10.5194/acp-5-1053-2005

[gh2147-bib-0030] Klimont, Z. , Kupiainen, K. , Heyes, C. , Purohit, P. , Cofala, J. , Rafaj, P. , Borken‐Kleefeld, J. , & Schöpp, W. (2017). Global anthropogenic emissions of particulate matter including black carbon. Atmospheric Chemistry and Physics, 17(14), 8681–8723. 10.5194/acp-17-8681-2017

[gh2147-bib-0031] Lamarque, J. F. , Emmons, L. K. , Hess, P. G. , Kinnison, D. E. , Tilmes, S. , Vitt, F. , Heald, C. L. , Holland, E. A. , Lauritzen, P. H. , Neu, J. , Orlando, J. J. , Rasch, P. J. , & Tyndall, G. K. (2012). CAM‐chem: Description and evaluation of interactive atmospheric chemistry in the Community Earth System Model. Geoscientific Model Development, 5(2), 369–411. 10.5194/gmd-5-369-2012

[gh2147-bib-0032] Lelieveld, J. , Evans, J. S. , Fnais, M. , Giannadaki, D. , & Pozzer, A. (2015). The contribution of outdoor air pollution sources to premature mortality on a global scale. Nature, 525(7569), 367–371. 10.1038/nature15371 26381985

[gh2147-bib-0033] Lelieveld, J. , Klingmüller, K. , Pozzer, A. , Burnett, R. T. , Haines, A. , & Ramanathan, V. (2019). Effects of fossil fuel and total anthropogenic emission removal on health and climate. Proceedings of the National Academy of Sciences, 116(15), 7192–7197. 10.1073/pnas.1819989116 PMC6462052 30910976

[gh2147-bib-0034] Liu, X. , Easter, R. C. , Ghan, S. J. , Zaveri, R. , Rasch, P. , Shi, X. , Lamarque, J. F. , Gettelman, A. , Morrison, H. , Vitt, F. , Conley, A. , Park, S. , Neale, R. , Hannay, C. , Ekman, A. M. L. , Hess, P. , Mahowald, N. , Collins, W. , Iacono, M. J. , Bretherton, C. S. , Flanner, M. G. , & Mitchell, D. (2012). Toward a minimal representation of aerosols in climate models: Description and evaluation in the Community Atmosphere Model CAM5. Geoscientific Model Development, 5(3), 709–739. 10.5194/gmd-5-709-2012

[gh2147-bib-0035] Lund, M. T. , Berntsen, T. K. , Heyes, C. , Klimont, Z. , & Samset, B. H. (2014). Global and regional climate impacts of black carbon and co‐emitted species from the on‐road diesel sector. Atmospheric Environment, 98, 50–58. 10.1016/j.atmosenv.2014.08.033

[gh2147-bib-0036] Masson‐Delmotte, V. , Zhai, P. , Pörtner, H.‐O. , Roberts, D. , Skea, J. , Shukla, P. R. , et al. (2018). Global warming of 1.5 °C. An IPCC special report on the impacts of global warming of 1.5 °C above pre‐industrial levels and related global greenhouse gas emission pathways, in the context of strengthening the global response to the threat of climate change. Report of the Intergovernmental Panel on Climate Change.

[gh2147-bib-0037] Matthes, S. , Grewe, V. , Sausen, R. , & Roelofs, G. J. (2007). Global impact of road traffic emissions on tropospheric ozone. Atmospheric Chemistry and Physics, 7(7), 1707–1718. 10.5194/acp-7-1707-2007

[gh2147-bib-0038] Miller, J , & Jin, L. (2018). Global progress toward soot‐free diesel vehicles in 2018. *ICCT ‐ International Council on Clean Transportation* Retrieved from https://theicct.org/sites/default/files/publications/Global_progress_soot_free_diesel_20180702.pdf

[gh2147-bib-0039] Miller, J. , Du, L. , & Kodjak, D. (2017). Impacts of world‐class vehicle efficiency and emissions regulations in select G20 countries. ICCT ‐ International Council on Clean Transportation. Retrieved from http://www.theicct.org/sites/default/files/publications/ICCT_G20-briefing-paper_Jan2017_vF.pdf

[gh2147-bib-0040] Morita, H. , Yang, S. , Unger, N. , & Kinney, P. L. (2014). Global health impacts of future aviation emissions under alternative control scenarios. Environmental Science and Technology, 48(24), 14659–14667. 10.1021/es5055379 25412200 PMC4270391

[gh2147-bib-0041] Myhre, G. , Shindell, D. , Bréon, F.‐M. , Collins, W. , Fuglestvedt, J. , Huang, J. , et al. (2013). Anthropogenic and natural radiative forcing. *Climate Change 2013:* The Physical Science Basis. Contribution of Working Group I to the Fifth Assessment Report of the Intergovernmental Panel on Climate Change, 659–740. 10.1017/CBO9781107415324.018

[gh2147-bib-0042] Niemeier, U. , Granier, C. , Kornblueh, L. , Walters, S. , & Brasseur, G. P. (2006). Global impact of road traffic on atmospheric chemical composition and on ozone climate forcing. Journal of Geophysical Research, 111, D09031 10.1029/2005JD006407

[gh2147-bib-0043] Rönkkö, T. , Kuuluvainen, H. , Karjalainen, P. , Keskinen, J. , Hillamo, R. , Niemi, J. V. , Pirjola, L. , Timonen, H. J. , Saarikoski, S. , Saukko, E. , Järvinen, A. , Silvennoinen, H. , Rostedt, A. , Olin, M. , Yli‐Ojanperä, J. , Nousiainen, P. , Kousa, A. , & Dal Maso, M. (2017). Traffic is a major source of atmospheric nanocluster aerosol. Proceedings of the National Academy of Sciences, 114(29), 7549–7554. 10.1073/pnas.1700830114 PMC5530662 28674021

[gh2147-bib-0044] Shindell, D. , Faluvegi, G. , Walsh, M. , Anenberg, S. C. , van Dingenen, R. , Muller, N. Z. , Austin, J. , Koch, D. , & Milly, G. (2011). Climate, health, agricultural and economic impacts of tighter vehicle‐emission standards. Nature Climate Change, 1(1), 59–66. 10.1038/nclimate1066

[gh2147-bib-0045] Silva, R. A. , Adelman, Z. , Fry, M. M. , & West, J. J. (2016). The impact of individual anthropogenic emissions sectors on the global burden of human mortality due to ambient air pollution. Environmental Health Perspectives, 124(11), 1776–1784. 10.1289/EHP177 27177206 PMC5089880

[gh2147-bib-0046] Stanaway, J. D. , Afshin, A. , Gakidou, E. , Lim, S. S. , Abate, D. , Abate, K. H. , Abbafati, C. , Abbasi, N. , Abbastabar, H. , Abd‐Allah, F. , Abdela, J. , Abdelalim, A. , Abdollahpour, I. , Abdulkader, R. S. , Abebe, M. , Abebe, Z. , Abera, S. F. , Abil, O. Z. , Abraha, H. N. , Abrham, A. R. , Abu‐Raddad, L. J. , Abu‐Rmeileh, N. M. E. , Accrombessi, M. M. K. , Acharya, D. , Acharya, P. , Adamu, A. A. , Adane, A. A. , Adebayo, O. M. , Adedoyin, R. A. , Adekanmbi, V. , Ademi, Z. , Adetokunboh, O. O. , Adib, M. G. , Admasie, A. , Adsuar, J. C. , Afanvi, K. A. , Afarideh, M. , Agarwal, G. , Aggarwal, A. , Aghayan, S. A. , Agrawal, A. , Agrawal, S. , Ahmadi, A. , Ahmadi, M. , Ahmadieh, H. , Ahmed, M. B. , Aichour, A. N. , Aichour, I. , Aichour, M. T. E. , Akbari, M. E. , Akinyemiju, T. , Akseer, N. , al‐Aly, Z. , al‐Eyadhy, A. , al‐Mekhlafi, H. M. , Alahdab, F. , Alam, K. , Alam, S. , Alam, T. , Alashi, A. , Alavian, S. M. , Alene, K. A. , Ali, K. , Ali, S. M. , Alijanzadeh, M. , Alizadeh‐Navaei, R. , Aljunid, S. M. , Alkerwi, A.'. , Alla, F. , Alsharif, U. , Altirkawi, K. , Alvis‐Guzman, N. , Amare, A. T. , Ammar, W. , Anber, N. H. , Anderson, J. A. , Andrei, C. L. , Androudi, S. , Animut, M. D. , Anjomshoa, M. , Ansha, M. G. , Antó, J. M. , Antonio, C. A. T. , Anwari, P. , Appiah, L. T. , Appiah, S. C. Y. , Arabloo, J. , Aremu, O. , Ärnlöv, J. , Artaman, A. , Aryal, K. K. , Asayesh, H. , Ataro, Z. , Ausloos, M. , Avokpaho, E. F. G. A. , Awasthi, A. , Ayala Quintanilla, B. P. , Ayer, R. , Ayuk, T. B. , Azzopardi, P. S. , Babazadeh, A. , Badali, H. , Badawi, A. , Balakrishnan, K. , Bali, A. G. , Ball, K. , Ballew, S. H. , Banach, M. , Banoub, J. A. M. , Barac, A. , Barker‐Collo, S. L. , Bärnighausen, T. W. , Barrero, L. H. , Basu, S. , Baune, B. T. , Bazargan‐Hejazi, S. , Bedi, N. , Beghi, E. , Behzadifar, M. , Behzadifar, M. , Béjot, Y. , Bekele, B. B. , Bekru, E. T. , Belay, E. , Belay, Y. A. , Bell, M. L. , Bello, A. K. , Bennett, D. A. , Bensenor, I. M. , Bergeron, G. , Berhane, A. , Bernabe, E. , Bernstein, R. S. , Beuran, M. , Beyranvand, T. , Bhala, N. , Bhalla, A. , Bhattarai, S. , Bhutta, Z. A. , Biadgo, B. , Bijani, A. , Bikbov, B. , Bilano, V. , Bililign, N. , Bin Sayeed, M. S. , Bisanzio, D. , Biswas, T. , Bjørge, T. , Blacker, B. F. , Bleyer, A. , Borschmann, R. , Bou‐Orm, I. R. , Boufous, S. , Bourne, R. , Brady, O. J. , Brauer, M. , Brazinova, A. , Breitborde, N. J. K. , Brenner, H. , Briko, A. N. , Britton, G. , Brugha, T. , Buchbinder, R. , Burnett, R. T. , Busse, R. , Butt, Z. A. , Cahill, L. E. , Cahuana‐Hurtado, L. , Campos‐Nonato, I. R. , Cárdenas, R. , Carreras, G. , Carrero, J. J. , Carvalho, F. , Castañeda‐Orjuela, C. A. , Castillo Rivas, J. , Castro, F. , Catalá‐López, F. , Causey, K. , Cercy, K. M. , Cerin, E. , Chaiah, Y. , Chang, H. Y. , Chang, J. C. , Chang, K. L. , Charlson, F. J. , Chattopadhyay, A. , Chattu, V. K. , Chee, M. L. , Cheng, C. Y. , Chew, A. , Chiang, P. P. C. , Chimed‐Ochir, O. , Chin, K. L. , Chitheer, A. , Choi, J. Y. J. , Chowdhury, R. , Christensen, H. , Christopher, D. J. , Chung, S. C. , Cicuttini, F. M. , Cirillo, M. , Cohen, A. J. , Collado‐Mateo, D. , Cooper, C. , Cooper, O. R. , Coresh, J. , Cornaby, L. , Cortesi, P. A. , & Cortinovis, M. (2018). Global, regional, and national comparative risk assessment of 84 behavioural, environmental and occupational, and metabolic risks or clusters of risks for 195 countries and territories, 1990‐2017: A systematic analysis for the Global Burden of Disease Stu. The Lancet, 392(10159), 1923–1994. 10.1016/S0140-6736(18)32225-6 PMC6227755 30496105

[gh2147-bib-0047] Stohl, A. , Aamaas, B. , Amann, M. , Baker, L. H. , Bellouin, N. , Berntsen, T. K. , Boucher, O. , Cherian, R. , Collins, W. , Daskalakis, N. , Dusinska, M. , Eckhardt, S. , Fuglestvedt, J. S. , Harju, M. , Heyes, C. , Hodnebrog, Ø. , Hao, J. , Im, U. , Kanakidou, M. , Klimont, Z. , Kupiainen, K. , Law, K. S. , Lund, M. T. , Maas, R. , MacIntosh, C. R. , Myhre, G. , Myriokefalitakis, S. , Olivié, D. , Quaas, J. , Quennehen, B. , Raut, J. C. , Rumbold, S. T. , Samset, B. H. , Schulz, M. , Seland, Ø. , Shine, K. P. , Skeie, R. B. , Wang, S. , Yttri, K. E. , & Zhu, T. (2015). Evaluating the climate and air quality impacts of short‐lived pollutants. Atmospheric Chemistry and Physics, 15(18), 10529–10566. 10.5194/acp-15-10529-2015

[gh2147-bib-0048] Tilmes, S. , Lamarque, J. F. , Emmons, L. K. , Kinnison, D. E. , Ma, P. L. , Liu, X. , Ghan, S. , Bardeen, C. , Arnold, S. , Deeter, M. , Vitt, F. , Ryerson, T. , Elkins, J. W. , Moore, F. , Spackman, J. R. , & Val Martin, M. (2015). Description and evaluation of tropospheric chemistry and aerosols in the Community Earth System Model (CESM1.2). Geoscientific Model Development, 8(5), 1395–1426. 10.5194/gmd-8-1395-2015

[gh2147-bib-0049] Turner, M. C. , Jerrett, M. , Pope, C. A. III , Krewski, D. , Gapstur, S. M. , Diver, W. R. , Beckerman, B. S. , Marshall, J. D. , Su, J. , Crouse, D. L. , & Burnett, R. T. (2016). Long‐term ozone exposure and mortality in a large prospective study. American Journal of Respiratory and Critical Care Medicine, 193(10), 1134–1142. 10.1164/rccm.201508-1633oc 26680605 PMC4872664

[gh2147-bib-0050] Turnock, S. T. , Allen, R. J. , Andrews, M. , Bauer, S. E. , Emmons, L. , Horowitz, L. , et al. (2020). Historical and future changes in air pollutants from CMIP6 models. Atmospheric Chemistry and Physics Discussions, 1–40. 10.5194/acp-2019-1211

[gh2147-bib-0051] Unger, N. , Bond, T. C. , Wang, J. S. , Koch, D. M. , Menon, S. , Shindell, D. T. , & Bauer, S. (2010). Attribution of climate forcing to economic sectors. Proceedings of the National Academy of Sciences, 107(8), 3382–3387. 10.1073/pnas.0906548107 PMC2816198 20133724

[gh2147-bib-0052] Unger, N. , Shindell, D. T. , & Wang, J. S. (2009). Climate forcing by the on‐road transportation and power generation sectors. Atmospheric Environment, 43(19), 3077–3085. 10.1016/j.atmosenv.2009.03.021

[gh2147-bib-0053] Wang, T. , Xue, L. , Brimblecombe, P. , Lam, Y. F. , Li, L. , & Zhang, L. (2017). Ozone pollution in China: A review of concentrations, meteorological influences, chemical precursors, and effects. Science of the Total Environment, 575, 1582–1596. 10.1016/j.scitotenv.2016.10.081 27789078

[gh2147-bib-0054] Xing, J. , Wang, S. X. , Jang, C. , Zhu, Y. , & Hao, J. M. (2011). Nonlinear response of ozone to precursor emission changes in China: A modeling study using response surface methodology. Atmospheric Chemistry and Physics, 11(10), 5027–5044. 10.5194/acp-11-5027-2011

[gh2147-bib-0055] Yan, F. , Winijkul, E. , Jung, S. , Bond, T. C. , & Streets, D. G. (2011). Global emission projections of particulate matter (PM): I. Exhaust emissions from on‐road vehicles. Atmospheric Environment, 45(28), 4830–4844. 10.1016/j.atmosenv.2011.06.018

[gh2147-bib-0056] Zhang, Q. , Jimenez, J. L. , Canagaratna, M. R. , Allan, J. D. , Coe, H. , Ulbrich, I. , Alfarra, M. R. , Takami, A. , Middlebrook, A. M. , Sun, Y. L. , Dzepina, K. , Dunlea, E. , Docherty, K. , DeCarlo, P. F. , Salcedo, D. , Onasch, T. , Jayne, J. T. , Miyoshi, T. , Shimono, A. , Hatakeyama, S. , Takegawa, N. , Kondo, Y. , Schneider, J. , Drewnick, F. , Borrmann, S. , Weimer, S. , Demerjian, K. , Williams, P. , Bower, K. , Bahreini, R. , Cottrell, L. , Griffin, R. J. , Rautiainen, J. , Sun, J. Y. , Zhang, Y. M. , & Worsnop, D. R. (2007). Ubiquity and dominance of oxygenated species in organic aerosols in anthropogenically‐influenced Northern Hemisphere midlatitudes. Geophysical Research Letters, 34, L13801 10.1029/2007GL029979

