# Supplementary material for: Global Climate and Human Health Effects of the Gasoline and Diesel Vehicle Fleets
Source: Geohealth. 2020 Mar 11;4(3):e2019GH000240. doi: 10.1029/2019GH000240 (PMC7065981; doi:10.1029/2019GH000240)
Supplement: Supplementary file 1 — Supporting Information S1 [file GH2-4-e2019GH000240-s001.docx]

**Supplemental Information for**

**Global climate and human health effects of the gasoline and diesel vehicle fleets**

Yaoxian Huang^1^, Nadine Unger^2^, Kandice Harper^3^ and Chris Heyes^4^

^1^Department of Civil and Environmental Engineering, Wayne State University, MI 48202, USA

^2^College of Engineering, Mathematics, and Physical Sciences, University of Exeter, Exeter, EX4 4QE, UK

^3^School of Forestry and Environmental Studies, Yale University, New Haven, CT 06511, USA

^4^International Institute for Applied Systems Analysis, Laxenburg, Austria

*Correspondence to*: Y. Huang, [yaoxian.huang@wayne.edu](mailto:yaoxian.huang@wayne.edu)

**Contents of this file**

Figures S1 to S5

**Figure S1.** Global spatial distribution of (a) shortwave (SW) and (b) longwave (LW) radiative effect at the top of atmosphere for the gasoline sector for year 2015, averaged over 2006-2010.

**
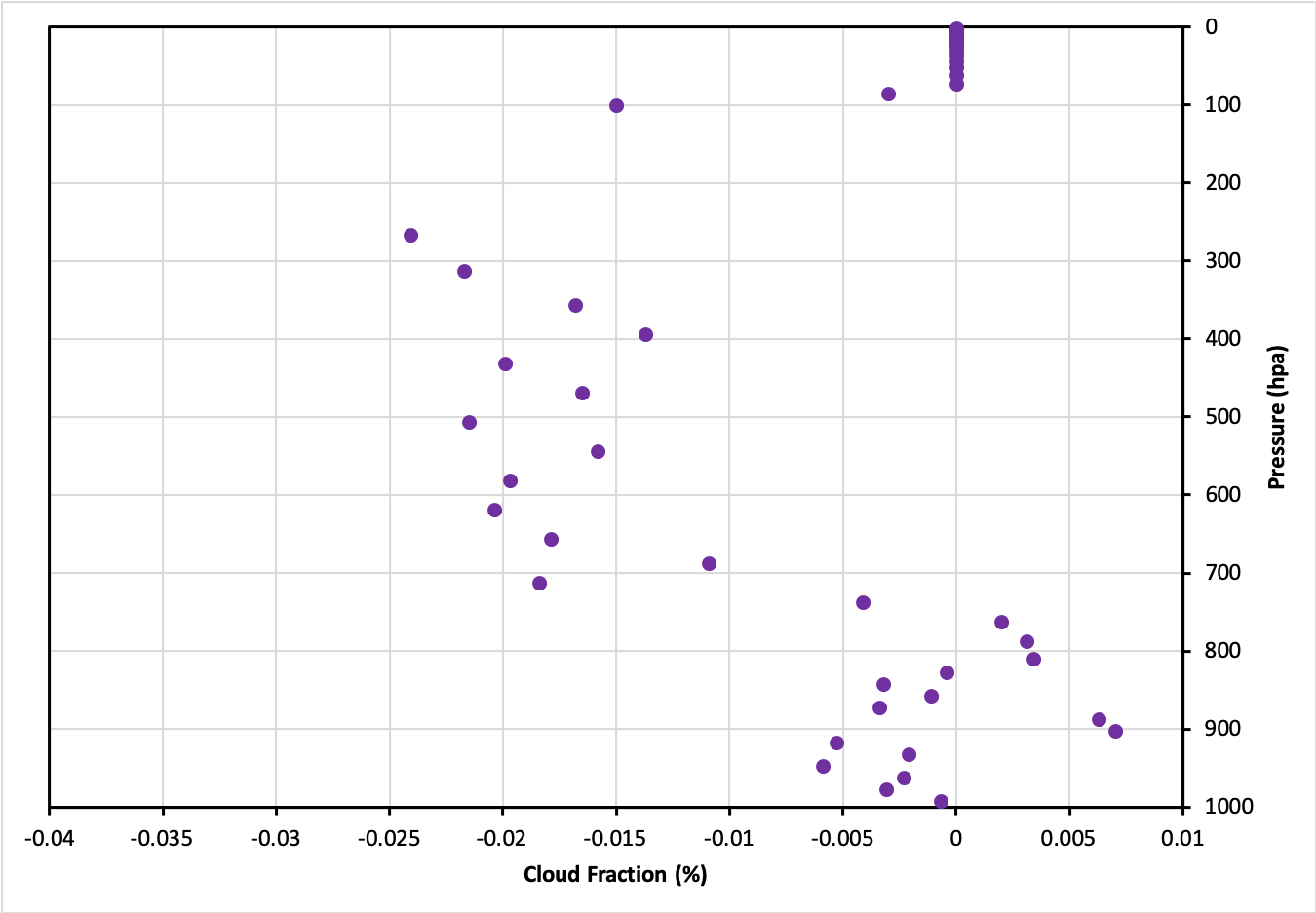
**

**Figure S2.** Changes in vertical profiles of cloud fractions from the gasoline sector averaged from 2006-2010 over the northern India Ocean, the area of which is defined as latitude 5°S-10°N, longitude 70°-100°E.

**Figure S3.** Same as Figure S1 but for the diesel sector.

**Figure S4.** Global annual mean spatial distribution of cloud fraction changes averaged from surface to middle troposphere (> 500 hPa) during the period of 2006-2010 for the gasoline (a) and diesel (b) sectors, respectively.

**Figure S5.** Changes in globally averaged vertical profile of cloud fractions for the diesel sector from 2006-2010.
